# A DPP6‐Targeted PET Tracer for Non‐Invasive Imaging of Transplanted Human Islets

**DOI:** 10.1111/dom.71343

**Published:** 2026-09-20

**Authors:** Amina Khalil, Arturo Roca‐Rivada, Svitlana Vasylovska, Lorenzo J. I. Balestri, Irina Velikyan, Sayro Jawurek, Alexandra C. Title, Burcak Yesildag, Luke R. Odell, Joey Lau, Decio L. Eizirik, Olof Eriksson

**Affiliations:** ^1^ Science for Life Laboratory, Department of Medicinal Chemistry Uppsala University Uppsala Sweden; ^2^ ULB Center for Diabetes Research, Medical Faculty Université Libre De Bruxelles Brussels Belgium; ^3^ Department of Medical Cell Biology Uppsala University Uppsala Sweden; ^4^ Department of Medicinal Chemistry Uppsala University Uppsala Sweden; ^5^ PET Center Uppsala University Hospital Uppsala Sweden; ^6^ InSphero AG Schlieren Switzerland

**Keywords:** beta cells, DPP6, imaging reagents, islet transplantation, PET, stem cell‐derived islets

## Abstract

**Introduction:**

Dipeptidyl peptidase‐like protein 6 (DPP6) is a potential molecular imaging biomarker for pancreatic and transplanted islets of Langerhans. 4hD29 is a single‐domain antibody with nanomolar affinity for DPP6. Here, we demonstrate the generation and validation of [^68^Ga]Ga‐NOTA‐4hD29 for Positron Emission Tomography (PET) imaging of human stem cell‐derived islets (SC‐islets) and human islets.

**Methods:**

The binding of fluorescently labelled 4hD29 to human islets, human beta cell models and pancreatic tissues from various species over time was characterised. NOTA‐4hD29 was radiolabelled with Gallium‐68, and the resulting tracer [^68^Ga]Ga‐NOTA‐4hD29 was evaluated for in vitro DPP6 binding as well as in vivo biodistribution. In vivo PET imaging was performed in immunodeficient mice transplanted with 400 to 800 SC‐islets or human islets, and the uptake signal was compared to non‐transplanted control mice.

**Results:**

Fluorescently labelled 4hD29 demonstrated specific binding and internalisation in human islets. Fluorescence signal remained detectable for up to 2 h following incubation, indicating sustained intracellular retention. [^68^Ga]Ga‐NOTA‐4hD29 bound to human islets with high specificity, and binding intensity correlated to islet purity. The binding of fluorescent and radiolabelled 4hD29 was negligible in extra‐pancreatic tissues. In vivo PET imaging enabled reliable detection of grafts comprised of 400 to 800 SC‐islets or human islets in immunodeficient mice.

**Conclusion:**

[^68^Ga]Ga‐NOTA‐4hD29 is a promising PET tracer for in vivo imaging of DPP6 in human islets. The tracer enabled non‐invasive detection of transplanted human islet grafts in the 400–800 IEQ range and demonstrated favourable biodistribution and dosimetry profiles, supporting its further development for islet imaging applications.

## Key Points

- Question.
  ○ The aim of the study was to validate Gallium‐68 labelled 4hD29, a PET tracer targeting DPP6, as a non‐invasive marker of human islets and stem cell‐derived islets.
- Pertinent findings.
  ○ [^68^Ga]Ga‐NOTA‐4hD29 bound to human islets with high specificity, and binding intensity correlated to islet purity. In vivo PET imaging enabled reliable detection of grafts comprised of as few as 400 islets in immunodeficient mice.
- Implications for patient care.
  ○ This technology can reliably and non‐invasively detect islets in living subjects and demonstrates a promising safety and dosimetry profile suitable for human use. Clinical translation is ongoing.

## 1 Introduction

The global rise in Type 1 diabetes (T1D), characterised by autoimmune destruction of pancreatic beta cells and consequent insulin deficiency, underscores the urgent need for accurate, non‐invasive methods to quantify beta cell mass, monitor its decline and assess preservation or restoration following emerging therapies [1, 2, 3, 4].

Islet transplantation using human islets has emerged as a potential cure for Type 1 diabetes by restoring insulin‐producing capability [5, 6]. More recently, this approach has advanced with the development of stem cell–derived islets (SC‐islets), including both induced pluripotent stem cells (iPSC) and embryonic stem cells (ES), which have shown promising engraftment, function and sustained insulin production in patients [7, 8]. However, the wide adoption of islet replacement therapies is hindered by limitations, including transplantation efficacy, islet supply and risks stemming from systemic immunosuppression. In this context, reliable monitoring of transplanted islets is crucial to inform therapeutic strategies and enhance clinical outcomes. Currently, beta cell function is assessed using indirect biomarkers such as C‐peptide levels, insulin secretion tests, fasting glucose and HbA1c [9], which do not directly reflect beta cell mass. Consequently, there is a strong need for molecular imaging approaches capable of directly and longitudinally visualising beta cell mass in vivo. Positron Emission Tomography (PET) is particularly well suited for this purpose due to its high sensitivity, allowing detection of femtomolar receptor concentrations [10].

Despite advancements in the field of PET radiopharmaceuticals, identifying imaging targets with sufficient islet specificity remains challenging [3, 11]. Several islet or beta cell‐specific receptors are in clinical evaluation as targets for PET tracers, including, for example, Glucagon Like Peptide‐1 Receptor (GLP‐1R) [12] and GPR44 [13]. However, no clinically validated molecular imaging marker for islets currently exists.

To address this problem, it is crucial to identify novel targets that offer enhanced specificity and sensitivity for imaging pancreatic islets. Dipeptidyl peptidase‐like protein 6 (DPP6), a Kv4‐potassium channel regulator, has recently been identified as a potential biomarker with a predominant expression in human pancreatic beta cells and, to a lesser extent, alpha cells [14]. This discovery led to the development of a high‐affinity camelid single‐domain antibody (sdAb, also referred to as a nanobody) that binds human DPP6 and was named 4hD29 (Figure S1A). To enable in vivo imaging of DPP6‐expressing islet cells, the 4hD29 sdAb was radiolabelled with a gamma emitter, Technetium‐99m. The tracer was evaluated in a murine model transplanted with varying quantities of primary human islets using Single Photon Emission Tomography (SPECT). Tracer signal intensity correlated with the number of transplanted islets (comparison between 1000 and 3000 islet equivalents, with only 3000 islet equivalents showing significant difference from basal level), demonstrating its potential as a quantitative marker of islet mass [15]. In the same study, the 4hD29 sdAb was adapted for PET imaging by radiolabelling with gallium‐68, and proof‐of‐concept PET imaging was demonstrated in mice bearing DPP6‐positive Kelly neuroblastoma xenografts [15]. However, PET imaging of transplanted human islets was not evaluated. Given the superior sensitivity and spatial resolution of PET compared with SPECT, PET technology has the potential to detect smaller and more dispersed islet grafts. In addition, PET generally results in lower radiation exposure to clinical subjects through the use of shorter‐lived radionuclides.

In the present study, we evaluated whether PET imaging with [^68^Ga]Ga‐NOTA‐4hD29 could improve the detection of smaller human islet and stem cell‐derived islet grafts. We further performed an extensive preclinical assessment of tracer specificity, biodistribution, dosimetry and graft detectability to support the development of DPP6‐targeted PET as a non‐invasive imaging approach for monitoring transplanted islets.

## 2 Methods

### 2.1 Culture of Human EndoC‐βH1 Cells, Human Islet Microtissues (hIsMTs) and HeLa Cells

The human pancreatic beta cell line EndoC‐βH1, human islet microtissues (hIsMTs) and HeLa cells were used for in vitro studies and maintained under standard culture conditions as previously described [16, 17]. Detailed descriptions of cell culture conditions, differentiation of induced pluripotent stem cells into (iPSC) islet‐like cells, RNA interference procedures and immunoblotting analyses are provided in the Supporting Informations.

### 2.2 4hD29 Conjugation With an Alexa Fluor 594 NHS Ester

4hD29 sdAb was conjugated to a fluorescence dye following a protocol adapted from Van Lith et al. [18]. The resulting fluorescent conjugate (Alexa‐4hD29) was purified and characterised prior to use.

Fluorescent labelling–based experiments, including immunocytochemistry, in vitro internalisation assays, microtissue labelling and longitudinal imaging and immunostaining of human multi‐tissue arrays and islets from different species, were performed using Alexa‐4hD29. Detailed staining procedures, incubation conditions, imaging parameters, quantification methods and antibody information are provided in the Supporting Informations and Table S1.

### 2.3 Gallium‐68 Radiolabelling of NOTA‐4hD29

The conjugation of p‐SCN‐Bn‐NOTA to 4hD29 and the radiolabelling of NOTA‐AhD29 are described in detail in the Supporting Informations. Briefly, NOTA‐AhD29 was labelled with Gallium‐68 using eluate from a ^68^Ga/^68^Ge generator according to standard methods.

### 2.4 In Vitro Autoradiography Binding Assay

An in vitro autoradiography assay was conducted using slides containing frozen sections of human islet fractions with varying purities (24%, 43% and 90% of islets), along with frozen sections of human embryonic stem cell‐derived islets (SC‐islets). Slides containing frozen sections of exocrine tissue served as negative controls. Slides containing frozen sections of human heart, liver and lung tissues were also included to investigate if the tracer bound to selected extra‐pancreatic tissues. The slides were incubated either with approximately 15 nM of [^68^Ga]Ga‐NOTA‐4hD29 alone or with a prior addition of 1 μM unlabelled 4hD29 protein for blocking. The incubation with the tracer was carried out in PBS containing 1% of BSA at room temperature for 60 min. Following incubation, the slides were washed three times in cold PBS and then dipped in Milli‐Q water to remove unbound tracer. Subsequently, the sections were air‐dried at 37°C for 10 min and then exposed to digital phosphorimager plates (BAS‐MS, Fujifilm) overnight. For autoradiogram quantification, a 10 μL reference droplet from the assay buffer containing radioactivity was included. The plates were then digitalized utilising a phosphorimager instrument (Amersham Typhoon FLA 9500 Phosphor Imager, GE Healthcare) with a pixel size of 50 μm. To verify the islet purity, the preparations were stained with dithizone (DTZ), a zinc‐chelating dye that selectively stains pancreatic islets red due to their high zinc content, as a part of the islet isolation process (Figure S2).

### 2.5 Animal Experiments

Animal experiments were performed to assess tracer biodistribution and binding in rats, immunodeficient mice and a pig. Male Sprague–Dawley rats (*n* = 11), NMRI nu/nu mice (*n* = 29) and one pig (crossbred Yorkshire × Swedish Landrace × Hampshire) were included. Detailed housing conditions, animal handling and experimental procedures are described in the Supporting Informations.

All animal procedures were approved by the Uppsala Animal Experiment Ethics Board and conducted in accordance with Uppsala University regulations (UFV 2007/724) and the ARRIVE guidelines.

### 2.6 Ex Vivo Biodistribution in Rats

Rats (*n* = 7, 346 ± 34 g) received [^68^Ga]Ga‐NOTA‐4hD29 (1.3 ± 0.6 nmol, 11.7 ± 5.0 MBq, molar activity of 9.2 ± 0.4 MBq/nmol). Animals were euthanized at 5, 30 or 60‐min post‐injection (*n* = 2, 2 and 3, respectively). Organs and tissues were collected, weighed and radioactivity was measured using a gamma counter. Uptake was normalised to body weight and injected activity and expressed as standardised uptake values (SUV). Blood samples were collected at each time point for plasma stability analysis.

### 2.7 In Vivo Biodistribution in Rats Using PET

Rats (*n* = 4, 297 ± 13 g) were intravenously injected with [^68^Ga]Ga‐NOTA‐4hD29 (1.4 ± 0.2 nmol, 4–12 MBq) via the tail vein under general anaesthesia (isoflurane 3.0% in a mixture of medical oxygen and air at a ratio of 50/50%, delivered at 450 mL/min). Animals were immediately positioned in a preclinical PET‐MRI system (nanoPET‐MRI, Mediso), and dynamic whole‐body PET data were acquired over 120 min. After PET imaging, a CT scan was performed using a preclinical SPECT/CT system (nanoSPECT/CT, Mediso) for anatomical reference and attenuation correction.

### 2.8 Access to Human Islets and Generation of SC‐Islets for in Vivo Transplantation

Human embryonic stem cells (hESCs; H1 cell line, WiCell) were differentiated into pancreatic islet‐like cells (SC‐islets) using a previously established seven‐stage protocol [19] incorporating specific modifications as described previously [20]. Detailed information, culture conditions, differentiation procedures and quality control are provided in the Supporting Informations.

### 2.9 PET/MRI of Mice Implanted With SC‐Islet or Human Islet Grafts

NMRI nu/nu mice with a weight of 23.5–30.9 g were transplanted intramuscularly with 400–800 islet equivalents (IEQ) of SC‐islets (*n* = 12) or human islets (*n* = 9); non‐transplanted mice served as controls (*n* = 8). After 7–8 weeks post‐transplantation, allowing for xenograft vascularization, each mouse received an intravenous injection of 7.0 ± 2.6 MBq of [^68^Ga]Ga‐NOTA‐4hD29 via the tail vein under general isoflurane anaesthesia and underwent dynamic whole‐body PET imaging for 60 min using a nanoPET‐MRI system (Mediso Medical Imaging Systems), followed by MRI for anatomical reference. After imaging, mice were euthanized, and grafts were collected for post‐mortem histological analysis. Immunohistochemical analysis and confocal imaging of explanted islet xenografts are described in the Supporting Informations.

### 2.10 PET Image Analysis

PET images were analyzed using PMOD Base Functionality (PBAS; PMOD Technologies LLC, Zurich, Switzerland). Manual segmentation of transaxial images was used to delineate organs and islet graft sites. Tracer uptake was quantified and expressed as standardised uptake values (SUV), corrected for injected activity, injection time and body weight. Uptake in islet grafts was quantified as the area under the curve (AUC) from 60‐min dynamic scans and expressed as the islet‐to‐muscle SUV ratio to account for background uptake.

### 2.11 Calculation of Human Predicted Dosimetry

The human predicted dosimetry of [^68^Ga]Ga‐NOTA‐4hD29 was extrapolated from in vivo PET biodistribution data in rats. Briefly, the residence time in each tissue was determined by calculating the area under the curve of the non‐decay corrected SUV curve over time, using the trapezoidal method. The uptake in tissues was normalised to a human model of organ sizes, summarised by Stabin and Siegler [21]. The absorbed doses at the organ level (mGy/MBq) as well as the whole‐body effective dose (mSv/MBq) were estimated using OLINDA/EXM. Annual organ or effective dose limits are based on Swedish regulatory guidelines for individuals > 18 years of age.

### 2.12 Statistics

All statistical analyses were performed using GraphPad Prism 10. The differences in tracer uptake between three or more groups, for example, the autoradiography assay or in vivo in transplanted compared to non‐transplanted control mice, were evaluated using ordinary one‐way ANOVA. A *p*‐value < 0.05 was considered statistically significant. Significance levels are indicated as follows: *p* < 0.05 (*), *p* < 0.01 (**), *p* < 0.001 (***) and *p* < 0.0001 (****).

## 3 Results

### 3.1 Alexa‐4hD29 Labelling Specificity

The fluorescence‐conjugated sdAb Alexa‐4hD29 was incubated at different concentrations with non‐DPP6‐expressing cells (HeLa cells; negative control) (Figure S3A), or DPP6‐expressing cells (EndoC‐βH1 cells) (Figure S3B), showing specific labelling of EndoC‐βH1 cells only. Signal intensity was parallel to sdAb concentration, and no signal was detected in HeLa cells incubated with the low or high sdAb concentrations. To assess Alexa‐4hD29 specific labelling of DPP6, EndoC‐βH1 cells were silenced for DPP6 by different concentrations of a specific siRNA. DPP6 efficient silencing was determined by immunoblot (Figure S3D). DPP6 silencing affected Alexa‐4hD29 signal in a concentration‐dependent manner, indicating that the fluorescence‐conjugated sdAb binds specifically to DPP6 (Figure S3C,E). To further characterise Alexa‐4hD29 sdAb binding to DPP6‐expressing cells, iPSC‐derived islet‐like cells were co‐stained with insulin and glucagon, along with Alexa‐4hD29. This showed that the sdAb co‐localises with insulin and, to a lesser extent, with glucagon‐expressing cells (Figure S3F).

### 3.2 In Vitro Alexa‐4hD29 Internalisation/Degradation

To assess internalisation and degradation of the sdAb by human beta cells, living EndoC‐βH1 cells (Figure 2A) and iPSC‐derived beta‐like cells (Figure 1B) were incubated at 37°C with Alexa‐4hD29 (50 μg/mL, 1 h). After washing, cells were maintained in culture and analysed at 0–6 h. Alexa‐4hD29 signal decayed progressively in both models, with approximately 50% of the signal remaining 2 h after exposure. To confirm these findings in human islets, human islet microtissues (hIsMTs) from a non‐diabetic donor were incubated with Alexa‐4hD29 (50 or 100 μg/mL, 2 h) and analysed at 0–96 h. Alexa‐4hD29 signal was still detectable at 24 h but returned to background levels by 48 h (Figures 1C and S4). Incubation with the sdAb did not affect microtissue health, morphology or internal organisation.

**FIGURE 1:**
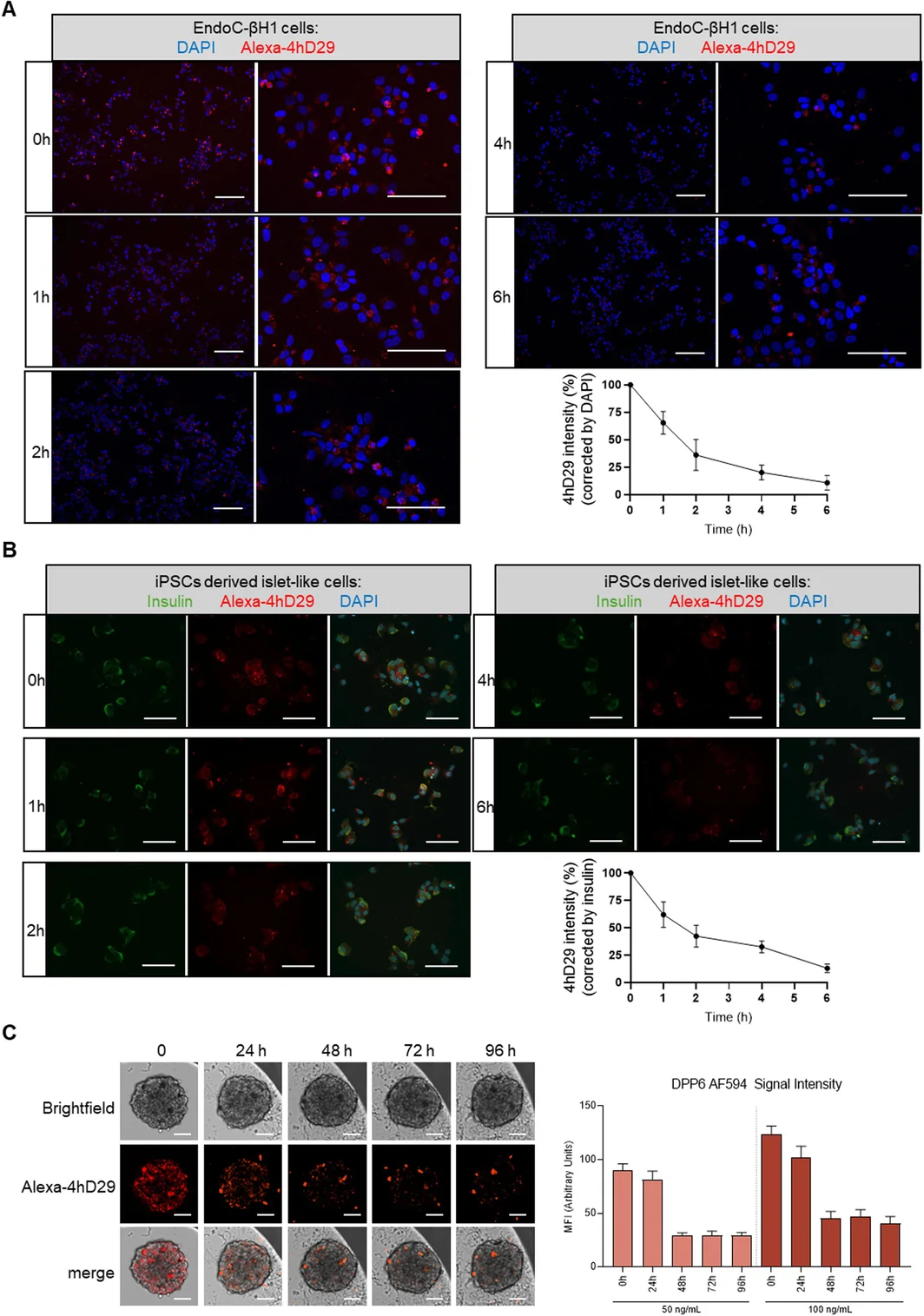
Time‐course of Alexa‐4hD29 internalisation and degradation in human beta‐cell models. (A) EndoC‐βH1 cells and (B) iPSC‐derived islet‐like cells were incubated with Alexa‐4hD29 (50 μg/mL) and analyzed at indicated time points by fluorescence microscopy. Signal intensity was normalised to DAPI (A) or insulin (B) and expressed as fold‐change relative to time 0. Data represent mean ± SEM of three independent experiments. (C) Human islet microtissues (hIsMTs) were incubated with Alexa‐4hD29 and imaged longitudinally up to 96 h; fluorescence intensity was background‐corrected using untreated controls. Representative images are shown.

### 3.3 Alexa‐4hD29 Degradation Pathway in Beta Cells

To characterise intracellular processing of 4hD29, living EndoC‐βH1 (Figure S2A) and iPSC‐derived islet‐like cells (Figure S4B) were incubated at 37°C with Alexa‐4hD29 (50 μg/mL, 1 h), washed, and maintained in culture for 1 or 4 h. Co‐localisation of Alexa‐4hD29 with LAMP1 (lysosomes), RAB7 (late endosomes) and RAB11 (recycling endosomes) was assessed.

Both beta cell models showed similar processing dynamics. After 1 h, approximately half of intracellular Alexa‐4hD29 localised to lysosomes (53% in EndoC‐βH1; 42% in iPSC‐derived cells), while 28% and 25% localised to late endosomes, respectively. Alexa‐4hD29 was also detected in recycling endosomes (26% and 18%), contributing to signal persistence. After 4 h, most remaining sdAb localised to degradation compartments (71% and 59%), with a smaller fraction persisting in recycling endosomes (37% and 29%).

### 3.4 In Vitro DPP6 Staining in Human Tissue Arrays and Islets From Different Species

Previous studies indicated that DPP6 is specifically expressed in human islets [3, 14, 15]. To independently confirm this, tissue microarrays comprising 59 human tissues from multiple donors were incubated with a commercial anti‐DPP6 antibody or with Alexa‐4hD29, revealing binding exclusively in human pancreas (Figures 2 and S5). Occasional weak signals observed in non‐pancreatic tissues were attributed to red blood cell autofluorescence (Figure S6).

**FIGURE 2:**
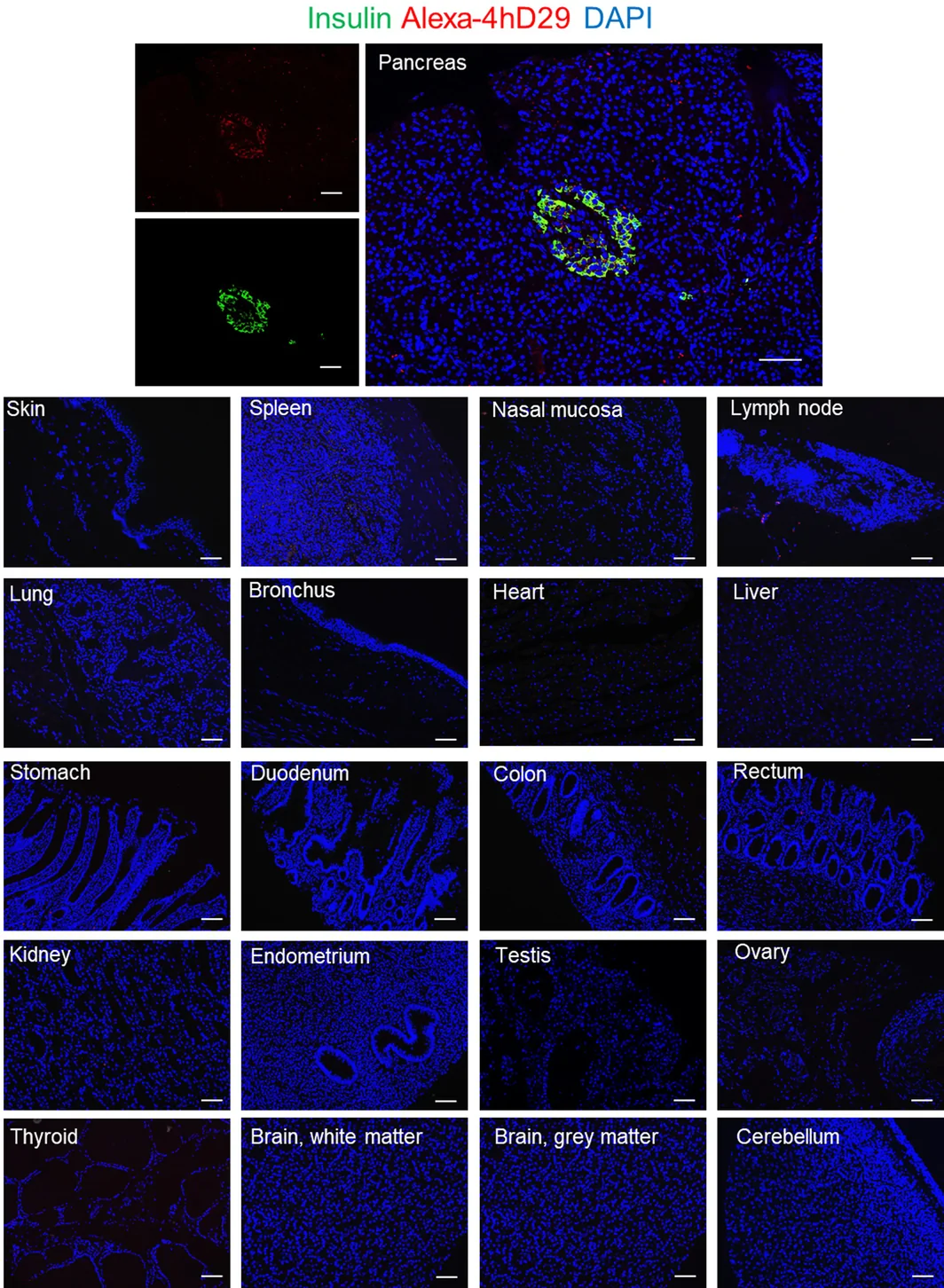
Alexa‐4hD29 signal is specific to human islets. Human Multi‐tissue Tissue MicroArrays corresponding to 59 human tissues from different locations obtained from different donors were incubated at 4°C overnight with Alexa‐4hD29 and insulin antibody. After incubation with the corresponding secondary antibody, slides were mounted with DAPI and representative images were analysed by fluorescence microscopy. The experiment was performed twice, and selected tissues are shown. Scale is 200 μm.

To identify a suitable preclinical species for PET dosimetry, toxicology and beta cell quantification, DPP6 expression was evaluated across species. As DPP6 is not expressed in rodents, pancreas sections from mouse, cynomolgus macaque and pig were analysed using a commercial anti‐DPP6 antibody, with human iPSC‐derived islet‐like cells as positive controls (Figure S7). Alexa‐4hD29 binding was observed in cynomolgus macaque islets but not in mouse or pig pancreas (Figures 3 and S8), identifying cynomolgus macaque as the appropriate species for translational PET studies.

**FIGURE 3:**
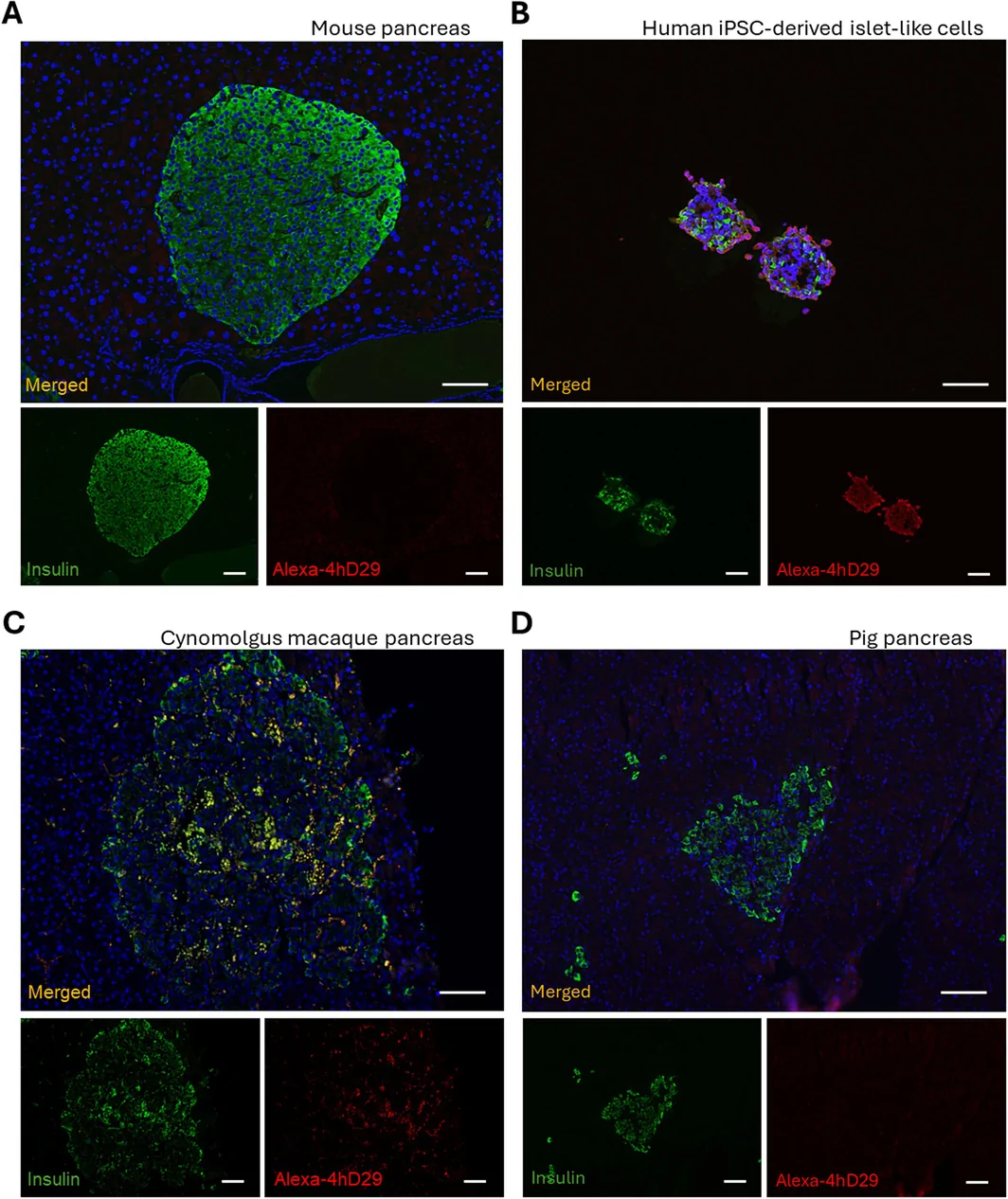
Alexa‐4hD29 signal is specific to primate islets. (A) Mouse pancreas, (B) human iPSC‐derived islet‐like cells, (C) Cynomolgus macaque pancreas or (D) pig pancreas were incubated at 4°C overnight with Alexa‐4hD29 and insulin antibodies. After incubation with the corresponding secondary antibody, slides were mounted with DAPI and representative images were analysed by fluorescence microscopy. The experiment was performed twice. Scale is 200 μm.

### 3.5 Conjugation of *p*‐SCN‐Bn‐NOTA to 4hD29

The desired Gallium‐68 chelating motif was attached through the formation of a covalent thiourea bond between the lysine residues of 4hD29 and the isothiocyanate functional group of the *p*‐SCN‐Bn‐NOTA. The reaction was conducted with a slight excess (12 equivalents) of the electrophile to minimise the risk of conjugation at multiple lysine residues. A specific pH of 8.5 was chosen to keep the lysine activated for the nucleophilic attack while simultaneously preventing the unwanted hydrolysis of isothiocyanate. The target compound was isolated by size‐exclusion chromatography and characterised by LC–MS analysis (Figures S9–S11).

### 3.6 Gallium‐68 Radiolabelling of NOTA‐4hD29

The radiolabelling of NOTA‐4hD29 resulted in a radiochemical yield (RCY) of 97.5% ± 0.5%. The radioactivity in the final product at the end of the synthesis was 56.5 ± 2.6 MBq with a radiochemical purity (RCP) of 99.8% ± 0.1%. The resulting molar activity at the end of the synthesis was typically around 10 MBq/nmol (11.48 ± 0.5 MBq/nmol).

### 3.7 In Vivo and In Vitro Stability of [ ^68^Ga]Ga‐NOTA‐4hD29

[^68^Ga]Ga‐NOTA‐4hD29 was stable in a formulation buffer for at least 60 min (Figure S12). Similarly, [^68^Ga]Ga‐NOTA‐4hD29 was stable in human plasma in vitro for the same time duration (Figures S12 and S13A,B). In vivo stability of [^68^Ga]Ga‐NOTA‐4hD29 in rat plasma was somewhat impacted, and approximately 65%–70% of radioactivity consisted of intact tracer 1 h after administration (Figures S12 and S13A–C).

### 3.8 In Vitro Autoradiography Binding Assay

[^68^Ga]Ga‐NOTA‐4hD29 binding was intense in preparations containing islets (Figure 4A). The highest uptake was observed in frozen sections of SC‐islets, with binding progressively decreasing as islet purity declined. Importantly, minimal background binding was observed in exocrine tissue. In contrast, blocking by pre‐incubation with 1 μM 4hD29 resulted in an almost complete abolishment of binding by [^68^Ga]Ga‐NOTA‐4hD29 (Figure 4B). The uptake of [^68^Ga]Ga‐NOTA‐4hD29 alone correlated closely with islet purity (*r* = 0.99, *R* ^2^ = 0.96, *p* < 0.001) (Figure 4C). After blocking, there was also a correlation with islet purity (*r* = 0.68, *R* ^2^ = 0.65, *p* < 0.01), but the slope was smaller than for [^68^Ga]Ga‐NOTA‐4hD29 alone (0.33 vs. 0.08, *p* < 0.0001). Furthermore, no binding of [^68^Ga]Ga‐NOTA‐4hD29 was detected in extra‐pancreatic tissues, including myocardium/heart, liver, spleen and lung tissue (Figure S15).

**FIGURE 4:**
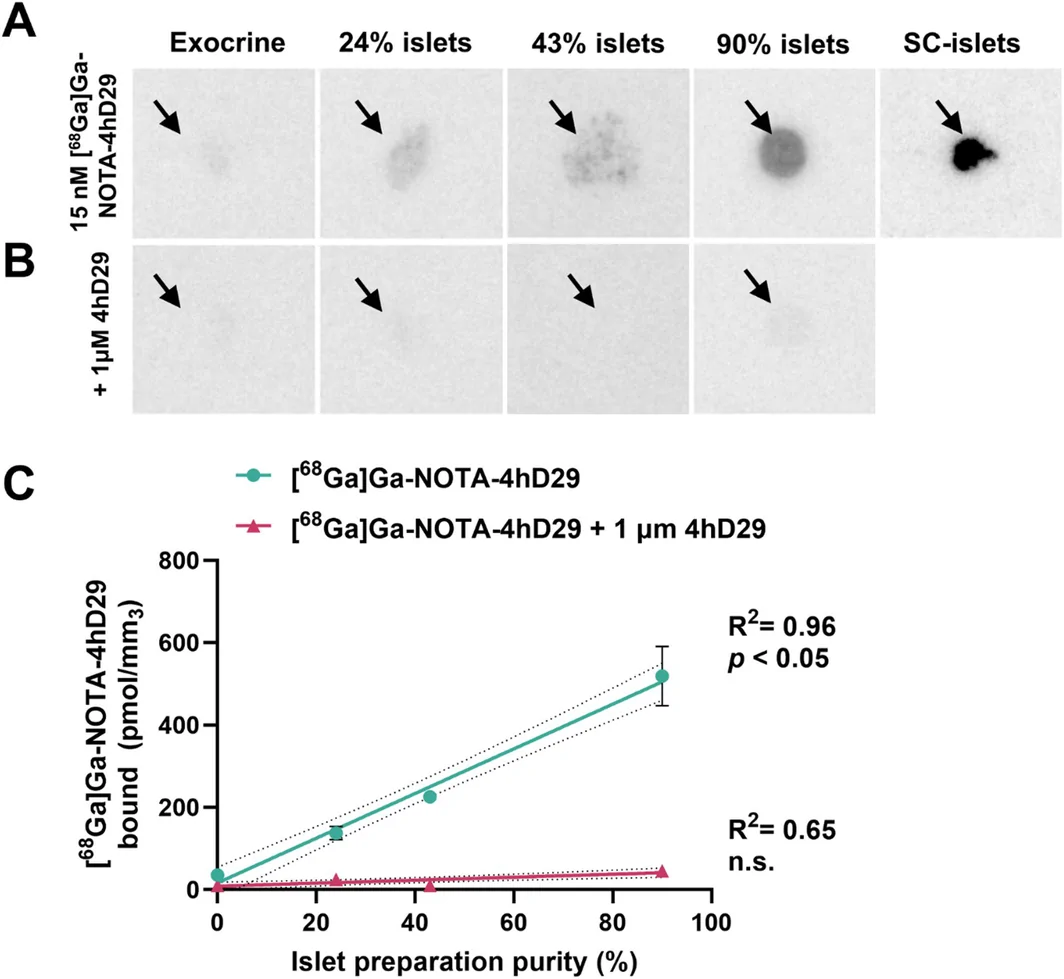
Autoradiograms of [^68^Ga]Ga‐NOTA‐4hD29 uptake in exocrine pancreatic tissue, islet preparations with increasing purity (24%, 43% or 90%) and pure stem cell‐derived islets (SC‐islets) (A). Co‐incubation with excess unlabelled 4hD29 blocks uptake, demonstrating high binding specificity (B). Black arrows indicate tissue locations. Quantification of [^68^Ga]Ga‐NOTA‐4hD29 binding and correlation with islet purity (C).

### 3.9 Ex Vivo and In Vivo Biodistribution in Rats

In healthy rats, the ex vivo biodistribution analysis of [^68^Ga]Ga‐NOTA‐4hD29 revealed predominant clearance through the kidneys and rapid washout from most tissues as well as circulation (Figure S16A). No relevant uptake or retention was observed in other organs.

In vivo rat PET/MRI confirmed these findings, demonstrating rapid renal excretion with early tracer accumulation in the bladder (Figure S16B,C). Circulating radioactivity was low at 60 min and nearly undetectable at 120 min post‐injection, indicating an optimal imaging window at these time points.

### 3.10 In Vivo Biodistribution in Pig

The biodistribution pattern seen in rat was confirmed in pig (see Supporting Informations for the study performance), which has a physiology closer to that of humans. [^68^Ga]Ga‐NOTA‐4hD29 was swiftly taken up in the kidneys, suggesting a predominant rapid renal clearance (Figure S17A). No significant tracer uptake was observed in any other organs except the kidneys, verifying low non‐specific background binding (Figure S17B).

### 3.11 PET/MRI of Mice With SC‐Islet or Human Islet Grafts

A PET signal originating from engrafted SC‐islets in the neck region was consistently detectable (Figure 5A), whereas no such uptake was observed in non‐engrafted control mice (Figure 5B). Uptake in mice engrafted with 400 or 800 IEQ SC‐islets was significantly higher than in controls (*p* < 0.05) (Figure 5C). PET signal intensity correlated with graft survival, as mice with graft loss showed uptake comparable to controls and significantly lower than mice with confirmed graft survival (Figure 5D).

**FIGURE 5:**
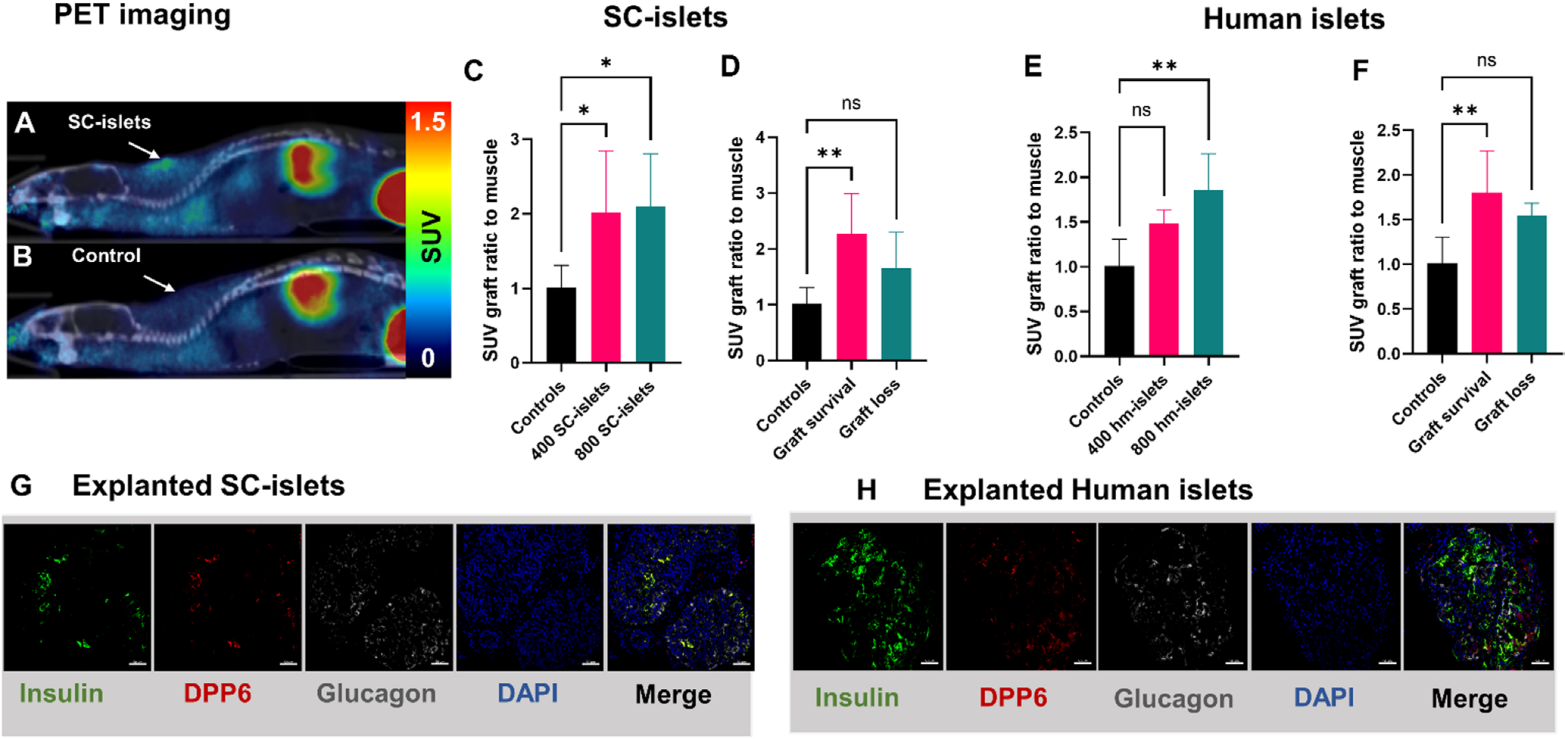
Representative sagittal PET/MRI image of [^68^Ga]Ga‐NOTA‐4hD29 signal in an SC‐islet graft in the neck muscle (A, white arrow) compared to non‐transplanted control (B, white arrow). (C, E) Quantification of PET signal in SC‐islet or human islet grafts (400 or 800 islets) versus controls. (D, F) Correlation between PET signal intensity and graft survival outcome. Uptake is expressed as the graft‐to‐muscle SUV ratio. (G, H) Representative post‐mortem histology of SC‐islet and human islet grafts confirming DPP6 immunostaining.

Similar results were obtained in mice engrafted with human islets. PET signal was increased in grafted animals compared with controls (Figure 5E) and correlated with islet viability assessed post‐mortem (Figure 5F). Histological analysis of SC‐islet and human islet grafts confirmed the presence of insulin‐positive cells and DPP6 expression (Figure 5G,H).

### 3.12 Dosimetry of [ ^68^Ga]Ga‐NOTA‐4hD29

Dosimetry extrapolation to human males estimates that the effective dose of [^68^Ga]Ga‐NOTA‐4hD29 is 0.025 mSv/MBq (Table S2). The individual limiting organ was the urinary bladder (0.41 mSv/MBq). Assuming an annual whole‐body effective dose limit of 10 mSv for young adults (often used by regulatory agencies), the present dosimetry of [^68^Ga]Ga‐NOTA‐4hD29 allows administration of approximately 400 MBq tracer per year in humans.

## 4 Discussion

Here, we present a comprehensive preclinical evaluation of the DPP6‐targeted PET tracer [^68^Ga]Ga‐NOTA‐4hD29 for imaging human islets. In vitro studies with Alexa‐4hD29 confirmed rapid and specific binding to the surface of living human beta cells, followed by internalisation and progressive degradation. These findings support a practical imaging window of approximately 60–120 min after tracer administration while limiting prolonged intracellular retention. Consistent with previous dose–response studies in EndoC‐βH1 cells [15], no evidence of impaired viability, morphology or tissue organisation was observed following exposure of human islet microtissues to 4hD29, supporting the biological compatibility of the tracer.

A major finding of the present study is that DPP6‐targeted PET imaging enabled detection of intramuscularly transplanted human and stem cell‐derived islet grafts containing 400–800 IEQ, a substantial improvement in sensitivity compared with the previous SPECT version of the 4hD29 tracer, for which reliable detection was demonstrated only for grafts containing approximately 3000 IEQ [14]. In addition, grafts exhibiting low tracer uptake in vivo were subsequently found to be negative for both insulin and DPP6 by immunohistochemistry, suggesting that DPP6 PET may provide information on graft viability. Importantly, both human islets and SC‐islets maintained robust DPP6 expression following engraftment. Furthermore, in vitro binding of [^68^Ga]Ga‐NOTA‐4hD29 correlated with islet number and islet purity (Figure 5C–F), supporting the quantitative nature of the tracer. While intramuscular transplantation is potentially a clinically relevant transplantation site, evaluation of [^68^Ga]Ga‐NOTA‐4hD29 PET in additional transplantation sites, such as the hepatic portal vein and omentum, will be an important next step to further establish its applicability across different clinical transplantation strategies [22]. Together, these findings indicate that DPP6‐targeted PET may provide a non‐invasive approach for assessing and monitoring transplanted human islet and SC‐islet grafts.

Several PET technologies are being explored to serve as non‐invasive imaging biomarkers of islet mass and survival. GLP‐1R targeting is probably the clinically most advanced, with Exendin‐4‐based PET tracers originally developed for improved localisation and diagnosis on insulinoma [23], but recently evaluated also in T1D [12], in Type 2 Diabetes (T2D) [24] and even in islet transplantation to the liver [25] or intramuscularly [26].

Despite promising results, there are still uncertainties regarding the specificity of GLP‐1R PET in the pancreas. Indeed, relatively high pancreatic binding (SUV_mean_ ≈ 3–5, depending on cohort) is seen in individuals with T1D (> 1 year post diagnosis, presumably very low beta cell mass) [12], similar to that seen in individuals with T2D (SUV_mean_ ≈ 4) without the need for exogenous insulin, indicating significant remaining beta cell mass [25]. Conversely, GLP‐1R expression can be downregulated under certain pathological conditions, such as chronic hyperglycemia, potentially leading to underestimation of beta cell mass [27]. Radiolabelled Exendin‐4 is also expected to bind many other extra‐pancreatic tissues since the GLP‐1R is also present in lungs, heart, gastrointestinal tract and pituitary gland [28, 29]. These observations highlight important biological differences between GLP‐1R‐ and DPP6‐targeted imaging. Accordingly, DPP6 and GLP‐1R represent distinct molecular targets with different expression profiles and may therefore provide complementary information depending on the specific imaging application and clinical context.

On‐target off‐tissue binding of targeting probes is normally not problematic for the delivery of imaging reagents, apart from additional deposition of radiation dose and potential ‘sinks’ for tracer accumulation. However, in the context of using targeting probes in broader applications such as therapeutic cargo delivery, for example, silencing RNAs (siRNAs) or other modulatory agents aimed at improving islet viability or function or modulating local islet immune environment [30, 31, 32, 33, 34]. The on‐target off‐tissue binding could result in unintended therapeutic exposure, including immune dysregulation, altered gene expression or toxicity in healthy organs, depending on the therapeutic cargo.

In this context, DPP6 represents an attractive imaging target. DPP6 is expressed in beta cells and, to a lesser extent, alpha cells, making it a marker of endocrine islet mass rather than beta‐cell mass alone [15]. This broader endocrine expression may improve sensitivity for graft detection while maintaining specificity for islet tissue. Bioinformatic analyses performed in the present study confirmed that DPP6 transcription within the pancreas is restricted to endocrine cells (Supporting Methods, Supporting Results, Figure S18A–C). Although DPP6 expression has been reported in neurons [32], the blood–brain barrier (BBB) effectively prevents access of circulating sdAbs to the central nervous system. Consistent with this, negligible brain uptake of ^[68^Ga]Ga‐NOTA‐4hD29 was observed in both rats (Figure S16B) and pigs (Figure S17), where negligible passage of [^68^Ga]Ga‐NOTA‐4hD29 over the BBB was observed at all time points after administration.

Furthermore, we demonstrated minimal binding of fluorescently labelled 4hD29 to a range of human tissues (Figure 3). Similarly, [^68^Ga]Ga‐NOTA‐4hD29 did not show appreciable binding to extra‐pancreatic tissues in the in vitro autoradiography assay (Figure S15).

Together, these observations support the highly restricted tissue distribution of DPP6 and its suitability as an imaging target and a candidate for islet‐targeted therapeutic delivery.

An important aspect of the extensive preclinical evaluation of the radiation exposure of the [^68^Ga]Ga‐NOTA‐4hD29 tracer. [^68^Ga]Ga‐NOTA‐4hD29 demonstrated a favourable preliminary dosimetry profile based on extrapolation from rat biodistribution data, with predicted organ doses remaining within acceptable limits. This feature is particularly relevant for longitudinal studies requiring repeated imaging, such as monitoring graft survival or disease progression. We estimate that administration of approximately 200 MBq per examination (≈2–3 MBq/kg, a realistic dose for an adult receiving a Gallium‐68 radiopharmaceutical) would allow at least two scans per year in research studies without exceeding accepted radiation exposure limits.

We also confirmed that DPP6 expression is restricted to primates and identified the Cynomolgus macaque as an appropriate species for future preclinical evaluation of [^68^Ga]Ga‐NOTA‐4hD29, including biodistribution, dosimetry, toxicology and PET imaging studies. Such studies will be important before considering first‐in‐human evaluation, particularly because the dosimetry estimates presented here are extrapolated from rat biodistribution data and should therefore be regarded as preliminary.

Several features of the tracer nevertheless support its continued preclinical development. The sdAb scaffold has been extensively evaluated in clinical settings as both a therapeutic and diagnostic platform and has generally demonstrated a favourable safety profile [35]. In addition, sdAbs can be produced recombinantly under Good Manufacturing Practice (GMP)‐compliant conditions. The use of the NOTA chelator is compatible with established clinical radiochemistry workflows, including automated generator‐based Gallium‐68 labelling procedures [36, 37]. Furthermore, the widespread availability of Gallium‐68 generators at nuclear medicine facilities could facilitate clinical implementation if the tracer is successfully validated in subsequent translational studies.

A limitation of the present study is the relatively small number of animals included in the biodistribution experiments, and these data should therefore be considered exploratory. Animal numbers were minimised in accordance with the Reduction principle of the 3Rs, and the experiments were designed primarily to characterise the overall biodistribution, clearance route and major organ uptake rather than to provide highly precise organ‐specific estimates.

In summary, the combined advantages of the in vitro and in vivo performance of sdAb 4hD29, the islet‐restricted expression of *DPP6* and the clinical suitability of Gallium‐68 labelling position this tracer as a highly promising tool for non‐invasive monitoring of islet grafts following transplantation, investigating islet biology in the context of diabetes and potentially guiding the delivery of islet‐targeted therapies.

## 5 Conclusion

This technology enables non‐invasive detection of transplanted human islet grafts in the 400–800 IEQ range, demonstrating a favourable biodistribution and dosimetry characteristics, supporting its further development for the assessment and longitudinal monitoring of transplanted islet grafts.

## Author Contributions

S.V., J.L., L.J.I.B., I.V., L.O., S.J., A.C.T., B.Y., O.K. and L.R.O. performed experimental research, interpreted data and edited the manuscript. A.K. and Arturo Roca‐Rivadaconceptualised and designed different parts of the study, performed experimental research, interpreted data and wrote the manuscript. O.E., J.L. and D.L.E. conceptualised the study, interpreted the data and wrote the manuscript. O.E. is the guarantor of this work and, as such, had full access to all the data in the study and takes responsibility for the integrity of the data and the accuracy of the data analysis.

## Funding

The study was funded by the Helmsley foundation (R‐2207‐05413) and Breakthrough Type 1 Diabetes (3‐SRA‐2026‐1848‐S‐B) for D.L.E. and O.E., by the Walloon Region (Win4Excellence grant, GT4Health project, agreement number 2310145) to D.L.E., by the Barndiabetesfonden (J.L. and O.E.), Diabetesfonden, Science for Life Laboratory, the Swedish Research Council (2020‐02312 and 2024‐03659; O.E.), Swedish Cancer Society/Cancerfonden (24 3754 Pj; O.E., 24 3889 Pj; L.R.O.), an EFSD/Lilly grant, StemTherapy (J.L.), ExoDiab and the Ernfors Family Fund (J.L. and O.E.).

## Ethics Statement

All the performed procedures were in accordance with the ARRIVE guidelines for animal research. The animal experiments were ethically reviewed, authorised by the Animal Ethics Committee of the Swedish Animal Welfare Agency and carried out in accordance with the European Directive 2010/63/EEC and the institutional guidelines at Uppsala University (UFV 2007/724). All human biological sample donors in Sweden provide informed consent that their donated tissues may be entered into a BioBank and used in medical research, pending review and approval by the Swedish Ethical Review Authority of individual studies. The use of human tissues and islets from Uppsala Biobank (registration #827) was approved by the Swedish Ethical Review Authority (2015/401, 2019/02790, 2024‐00969) and were anonymized, collected, and treated according to local institutional and Swedish national rules and regulations. The use of human blood from the local biobank was approved by the Swedish Ethical Review Authority (2024‐05050). The differentiation and use of iPSCs into islet‐like cells was approved by the Ethics Committee of the Erasmus Hospital, Université Libre de Bruxelles, reference P2019/498.

## Conflicts of Interest

O.E. is a part‐time employee of Antaros Tracer AB. S.J. and A.C.T. are employees of InSphero AG, a company commercialising human islet microtissues and related services. B.Y. is a member of the management team of InSphero AG. D.L.E. is a member of the Scientific Advisory Board of InSphero AG. D.L.E. has a patent on the use of nanobodies targeting DPP6 for human beta cell imaging. The other authors declare no conflicts of interest.

## Supporting information


**Figure S1:** Schematic illustration of the original DPP6 targeting sdAb (A), chelator conjugation to generate the precursor peptide (B) as well as Gallium‐68 radiolabelling conditions to form the PET tracer [^68^Ga]Ga‐NOTA‐4hD29 (C).
**Figure S2:** Representative dithizone staining microscopy images for the islet preparations with 24%, 43% or 90% of beta cell purity, with islets (zinc‐containing cells) appearing red and exocrine cells in yellow.
**Figure S3:** Characterisation of Alexa‐4hD29 binding to human beta cells. (A) HeLa cells were incubated with different concentrations of Alexa‐4hD29 (100 or 6.25 μg/mL). Images were obtained by fluorescence microscopy. Images are representative of three independent experiments. (B) EndoC‐βH1 cells were incubated with different concentrations of Alexa‐4hD29 (100, 50, 25 or 6.25 μg/mL). Images were obtained by fluorescence microscopy. Images are representative of three independent experiments. (C) EndoC‐βH1 cells were silenced for DPP6 by using different concentrations of DPP6‐specific siRNA (7.5, 15 or 30 nM). After 48 h of recovery the cells were fixed and incubated with Alexa‐4hD29. Images were obtained by fluorescence microscopy. Images are representative of three independent experiments. (D) DPP6 and GAPDH protein levels were analysed by immunoblot. Results are means ± SEM of three independent experiments. Bands were quantified by densitometry and corrected by GAPDH. Results are presented as fold‐variation compared to untreated cells, considered as 1. *p < 0.05, **p < 0.01 and ***p < 0.001 versus untreated cells. One‐way Anova. (E) DPP6 versus DAPI relative intensity comparison. Results are presented as fold‐variation compared to untreated cells, considered as 1. **p < 0.01, and ***p < 0.001 versus untreated cells. One‐way Anova. (F) Dispersed iPSC‐derived islet‐like cells were incubated with Alexa‐4hD29 plus insulin and glucagon. Images were obtained by fluorescence microscopy. Images are representative of three independent experiments. Scale is 200 μm.
**Figure S4:** Autofluorescence of Human islet microtissues (hIsMTs). HIsMTs were incubated at 37°C in the absence of Alexa‐4hD29. Cells were then washed three times with PBS and kept in culture at 37°C at different time‐points (0, 24, 48, 72 or 96 h). Images are representative of a single islet organoid. Scale is 50 μm.
**Figure S5:** Alexa‐4hD29 enters the beta cell lysosomal pathway. Living EndoC‐βH1 (A) or iPSC‐derived islet‐like cells (B) were incubated at 37°C with 50 μg/mL Alexa‐4hD29 for 1 h. Cells were the washed three times with PBS and kept in culture 1 h or 4 h at 37°C. After this, cells were fixed, and preparations were incubated with antibodies recognising LAMP1, RAB7 or RAB11. Preparations were then mounted with DAPI for widefield fluorescence microscopy. Scale is 200 μm. Results are means ± SEM of three independent experiments.
**Figure S6:** Red blood cells' autofluorescence. Human Multi‐tissue Tissue MicroArrays corresponding to 59 human tissues from different locations obtained from different donors were incubated or not at 4°C overnight with Alexa‐4hD29 and insulin antibody, or with PBS. After incubation with the corresponding secondary antibody, slides were mounted with DAPI and representative images were analysed by fluorescence microscopy. Images on the left correspond to consecutive slide cuts incubated with PBS; on the right, consecutive slide cuts incubated with antibodies. The experiment was performed twice and the selected tissues are shown. Scale is 200 μm.
**Figure S7:** DPP6 signal is specific to human islets. (a) Human Multi‐tissue Tissue MicroArrays corresponding to 59 human tissues from different locations obtained from different donors were incubated at 4°C overnight with DPP6 and insulin antibodies. After incubation with the corresponding secondary antibodies, slides were mounted with DAPI and representative images were analysed by fluorescence microscopy. The experiment was performed twice, and selected tissues are shown. Scale is 200 μm.
**Figure S8:** DPP6 signal is specific to primate beta cells. (A) Mouse pancreas, (B) human iPSC‐derived islet‐like cells, (C) Cynomolgus macaque pancreas or (D) pig pancreas were incubated at 4°C overnight with DPP6 and insulin antibodies. After incubation with the corresponding secondary antibodies, slides were mounted with DAPI and representative images were analysed by fluorescence microscopy. The experiment was performed twice. Scale is 200 μm.
**Figure S9:** Representative LC–MS trace of the coupling reaction between the 4hD29 nanobody and *p*‐SCN‐Bn‐NOTA (ratio of 1:12) after 22 h of incubation at room temperature.
**Figure S10:** Representative LC–MS trace for the collected fraction 4 showing the successful purification of the NOTA‐conjugated 4hD29 using a PD‐10 desalting column.
**Figure S11:** Representative LC–MS trace for the collected fraction 6 showing the successful purification of the NOTA‐conjugated 4hD29 using a PD‐10 desalting column.
**Figure S12:** The stability of [^68^Ga]Ga‐NOTA‐4hD29 in formulation buffer, in vitro in human plasma, or in vivo in rat plasma, expressed as the percentage of intact tracer over time.
**Figure S13:** HPLC radio‐chromatograms showing the stability of [^68^Ga]Ga‐NOTA‐4hD29 in vitro in human plasma after 15 min (A) or 60 min (B) incubation.
**Figure S14:** Representative HPLC radiochromatograms showing the stability of [^68^Ga]Ga‐NOTA‐4hD29 in vivo in rat plasma after 5 min (A), 30 min (B) or 60 min (C) after tracer injection.
**Figure S15:** Representative autoradiograms demonstrating low or negligible binding of [^68^Ga]Ga‐NOTA‐4hD29 to spleen, heart (myocardium), liver and lung.
**Figure S16:** Ex vivo biodistribution in rat demonstrating the retention of [^68^Ga]Ga‐NOTA‐4hD29, expressed as SUV (A). Representative PET/MRI images illustrating the biodistribution of [^68^Ga]Ga‐NOTA‐4hD29 in vivo in rat at 2, 60 or 120 min post‐ injection (B). Quantification of the in vivo uptake in rats as examined by PET imaging for up to 120 min (C). Br: brain, Li: liver, Lu: lungs, Ki: kidney.
**Figure S17:** Biodistribution of [^68^Ga]Ga‐NOTA‐4hD29 in pig visualised through representative dynamic PET/CT scanning at 15, 30 and 60 min post‐tracer injection. (A). Quantification of PET uptake data as standardised uptake values (SUV) (B) Li: liver, Lu: lungs, Ki: kidney.
**Figure S18:** Single‐cell RNA sequencing data from human pancreas, showing annotated clusters of distinct pancreatic and immune cell types (A). UMAP visualisation of *DPP6* expression, with a gradient from low (green) to high (purple) transcription (B). Histogram showing *DPP6* expression levels in individual endocrine subtypes (alpha, beta, delta, PP cells) (C).
**Table S1:** List of primary and secondary antibodies used.
**Table S2:** Organ and whole‐body human predicted dosimetry of [^68^Ga]Ga‐NOTA‐4hD29, extrapolated from rat in vivo biodistribution data. Text in red indicates limiting annual doses and potential dosing limits based on national Swedish regulations.

## Data Availability

The datasets used and/or analyzed during the current study are available from the corresponding authors on reasonable request.
