# Supplementary material for: A DPP6‐Targeted PET Tracer for Non‐Invasive Imaging of Transplanted Human Islets
Source: Diabetes Obes Metab. 2026 Sep 20;28(12):11676–88. doi: 10.1111/dom.71343 (PMC13731066; doi:10.1111/dom.71343)
Supplement: Supplementary file 1 [file DOM-28-11676-s001.docx]

**Supplementary Materials**

**A DPP6-targeted PET tracer for non-invasive imaging of transplanted human islets**

Amina Khalil and Arturo Roca-Rivada et al.

**Supplementary Methods**

*Culture of human EndoC-βH1 cells, Human islets microtissues (hIsMTs), and HeLa cells*

EndoC-βH1 cells were free of mycoplasma infection, as determined by monthly testing using the MycoAlert Mycoplasma Detection kit (Lonza, Basel, Switzerland).

Human islet microtissues (hIsMTs) (InSphero, Schlieren, Switzerland, MT-04-002-01-60) were generated after dispersion and reaggregation of primary human islets from a single donor (UNOS ID #AMCF155; female; 44y.o.; Hispanic; BMI 22.45; positive serologies: CMV IgG; HbA1c: 5.5%; cause of death: stroke). HIsMTs were cultured in standard culture medium (InSphero, CS-07-005-01).

Human wild-type HeLa cell line was purchased from Abcam (Abcam, Cambridge, UK). Cells were grown in 10 ml DMEM/High Glucose supplemented with 10% FBS and 2% penicillin–streptomycin (Thermo Fisher Scientific, Waltham, MA, USA) at 37 °C and 5% CO2. HeLa cells were free of mycoplasma infection, as determined by monthly testing using the MycoAlert Mycoplasma Detection kit (Lonza).

*Differentiation of induced pluripotent stem cells (iPSC) into islet-like cells for in vitro studies*

The human iPSC line 1023A derived from skin fibroblasts was kindly provided by DM Egli (Columbia University, NY, USA). Cells were cultured in Matrigel-coated plates (Corning, NY, USA) in E8 medium (Invitrogen Life Technologies, Paisley, UK) and passaged with 0.5 mmol/l EDTA (Invitrogen Life Technologies) twice per week. Cell quality and pluripotency were monitored using the MycoAlert Mycoplasma Detection kit for mycoplasma infection, cell karyotyping (Bio.be, Brussels, Belgium) for chromosomal abnormalities and immunocytochemical staining for pluripotency markers were performed as previously described (1). Islet-like cell differentiation was performed by following a seven-step protocol previously published and validated by our group (2-4). After differentiation, cell aggregates were included in paraffin, or dispersed, seeded on Matrigel-coated culture plates and cultured in HAM’s F-10 medium (Thermo Fisher Scientific) containing 2% fatty acid-free BSA (Roche, Basel, Switzerland), 2 mmol/l GlutaMAX (Thermo Fisher Scientific), 50 µM IBMX, and 100 U/ml penicillin–streptomycin (Thermo Fisher Scientific) for cell imaging analysis.

*RNA interference*

EndoC-βH1 cells were transfected overnight with 7.5, 15, or 30 nmol/l siRNA; after changing the medium to their corresponding standard fresh medium, cells were left to recover for 48 h. siRNA targeting DPP6 was previously validated by our group (siDPP6; 5′-CACGGATGAGGGAGTACTACA-3′; SI04656701; Qiagen, Venlo, the Netherlands). Transfection was performed using Lipofectamine RNAiMax (Invitrogen Life Technologies) as described (4). Allstars Negative Control siRNA (siCTRL; Qiagen) was used as a negative control.

*Immunoblot*

Total protein was extracted using Laemmli or RIPA buffer supplemented with phosphatase and protease inhibitors (Roche) and separated on 10% SDS–PAGE. Nitrocellulose membranes were probed using specific primary antibodies diluted in TBST (TBS, 0.1% Tween 20) with 5% BSA. After overnight incubation at 4°C, membranes were probed for 1 h at room temperature with peroxidase-conjugated secondary antibodies diluted 1:5000 in TBST with 5% BSA. Detection of immunoreactive bands was performed using a chemiluminescent substrate (SuperSignal West Femto, Thermo Fisher Scientific) using a FUSION FX system (Vilber, Marne-la-Vallée, France). The densitometric values were quantified by ImageJ (5) and normalized to GAPDH after background subtraction. Antibody references and dilutions are listed in Supplementary Table 1.

*4hD29 conjugation with an Alexa Fluor 594 NHS Ester*

Briefly, 4hD29 was concentrated and dialysed to metal-free PBS. Then, pH was adjusted to 8.5 by adding 1:10 v/v 1.0M metal-free pH 8.5 NaHCO_3_ and incubated for 1h at RT with 0.5 mg of Alexa Fluor 594 NHS Ester (Thermo Fisher Scientific). The conjugated sdAb was then dialysed to 0.25 M metal-free ammonium acetate buffer pH 5.5 to remove the non-labeled dye. The final product was named Alexa-4hD29 in the subsequent experiments. Efficiency of the conjugation and the optimal concentrations for the conjugated sdAb were assayed and settled prior to the different experiments described in Results.

*Immunocytochemistry*

EndoC-βH1 cells, HeLa cells, or iPSC-derived islet-like cells were fixed in 4% paraformaldehyde for 15–20 min. After permeabilization with 0.5% triton-X100 in PBS, cells were blocked with UltraV block (Thermo Fisher Scientific) for 10 min and then incubated with primary antibodies diluted in 0.1% Tween in PBS overnight at 4°C. Cells were then washed with PBS and incubated with secondary antibodies diluted in 0.1% Tween in PBS. Samples were mounted with Vectashield with DAPI (Vector Laboratories, Newark, CA, USA) and covered with glass coverslips. The antibodies are listed in Supplementary Table 1. Images were acquired by widefield fluorescence microscopy (Carl Zeiss Microscopy GmbH, Jena, Germany). In the experiments in which living cells were incubated with Alexa-4hD29 (50 μg/ml), cells grown on μ-Slide 18-well Glass Bottom plates (ibidi, Gräfelfing, Germany) were immediately fixed in 4% paraformaldehyde for 15–20 min, washed with PBS, and stained with DAPI (Vector Laboratories).

*In vitro internalization*

To assess the internalization/degradation timing of the sdAb, living EndoC-βH1 cells or iPSC-derived islet-like cells grown on μ-Slide 18-well Glass Bottom plates (ibidi) were incubated at 37ºC with 50 μg/ml Alexa-4hD29 for 1h. Cells were then washed three times with PBS and kept in culture at 37ºC at different time points (0, 1h, 2h, 4h, or 6h). After this, cells were fixed, and the preparations were stained with DAPI (Vector Laboratories) for fluorescence microscopy after incubation with primary and secondary antibodies, if required. Alexa-4hD29 signal was corrected by DAPI or insulin expression, as specified in the figure legends. Images were acquired by widefield fluorescence microscopy (Carl Zeiss Microscopy GmbH).

*Microtissue culture and labelling with Alexa-4hD29*

Human islet microtissues (hIsMTs) (InSphero, MT-04-002-01-60) were reaggregated for 5 days in Akura hanging-drop plates (InSphero, CS-PF24). After that, they were transferred to Akura 96w plates (InSphero, CS-09-001-00). hIsMTs were cultured in human islet maintenance medium (InSphero, CS-005-01) for 6 days, medium was exchanged every 2-3 days. hIsMTs were incubated with the Alexa-4hD29 (50 μg/mL or 100 μg/mL) for 2 hours. hIsMTs were washed 3x with medium, transferred to a fresh Akura 96w plate and washed 1 more time. Images were taken with Yokogawa CQ1 benchtop system (Yokogawa GesmbH, Amersfoort, Netherlands) every 24 hours for 5 days. Medium was exchanged every 2-3 days.

*In vitro immunostaining of human tissue arrays and islets from different species*

Two sets of Human Multi-tissue Tissue MicroArrays were purchased from Bio-Techne (Minneapolis, MN, USA; reference: NBP2-78113), consisting of cuts of 59-60 paraffin blocks of human tissues from different organs, tissues and donors. In parallel, paraffin-included sections of iPSC-derived islets and paraffin-included pancreatic sections of mouse, cynomolgus macaque and pig were used to screen for DPP6 and 4hD29 binding specificity by fluorescent immunohistochemistry (IHC). Briefly, slides were dewaxed in xylene and rehydrated with a gradient of ethanol and distilled water. Samples were then microwaved for 10 minutes for antigen retrieval and blocked for 1h in UltraCruz blocking reagent (Santa Cruz Biotechnology, Dallas, TX, USA). After that, slides were incubated overnight with primary antibodies against insulin and commercial anti-DPP6 diluted in 0.1% Tween in PBS, or with 100 μg/ml Alexa-4hD29 diluted in 0.1% Tween in PBS. Slides were then washed with PBS and incubated with secondary antibodies diluted in 0.1% Tween in PBS. Slides were mounted with Vector TrueVIEW Autofluorescence Quenching Kit with DAPI (Vector Laboratories) and covered with glass coverslips. Images were acquired by fluorescence microscopy (Carl Zeiss Microscopy GmbH). Consecutive slides were incubated in the absence of antibodies for autofluorescence as a negative control. Antibodies used are listed in Supplementary Table 1.

*Conjugation of p-SCN-Bn-NOTA to 4hD29*

The coupling of the 4hD29 sdAb with 2-S-(4-Isothiocyanatobenzyl)-1,4,7-triazacyclononane-1,4,7-triacetic acid (p-SCN-Bn-NOTA) was carried out using a protocol adapted from Demine et al (Supplementary Figure 1B) (6). To begin, the 4hD29 (1.1 mg/mL) was buffer-exchanged using a 0.05 M Na_2_CO_3_ buffer at pH 8.5 with a PD-10 Desalting column (GE Healthcare, Yvelines, France). The fractions containing the highest concentrations of 4hD29 were collected and used for the next step.

Next, p-SCN-Bn-NOTA (0.6 mg, 0.98 µmol) was added to achieve a molar ratio of 1:12 for the 4hD29 sdAb to p-SCN-Bn-NOTA. The pH was adjusted to 8.5 to facilitate the reaction, which was then stirred at room temperature (RT) overnight. After a 22-hour incubation at RT, the reaction was stopped by lowering the pH to 5.7 using 1M HCl.

To remove the unconjugated NOTA from the solution, ultracentrifugation was performed using a 0.1 M CH_3_CO_2_Na buffer at pH 5.5 in a Vivaspin tube (Sartorius Stedium Lab, Stonehouse, Gloucestershire, UK), at 1820 g for 6 minutes at 15°C. At this stage, the mixture was ready for size exclusion chromatography (SEC) via a PD-10 desalting column. After equilibrating the column with 0.1 M CH_3_CO_2_Na, the NOTA-4hD29 was loaded and eluted with this buffer. The fractions with the highest purity and concentrations were collected (see LC-MS data in Supplementary Figures 2-4).

*Gallium-68 radiolabeling of NOTA-4hD29*

Before the radiolabeling procedure (3-4 h), the ^68^Ga/^68^Ge generator (IGG100, Eckert and Ziegler, Berlin, Germany) was pre-eluted with a 0.1 M metal-free hydrochloric acid solution (HCl). For the radiolabeling procedure, the generator was eluted with 5 mL of metal-free HCl. The first fraction (1 mL) with the lowest activity was discarded. The second fraction of 1 mL with the highest activity was used for the labeling, and the rest was eluted into the waste. The pH of the eluent was adjusted to pH 4.5 using sodium acetate buffer and sodium hydroxide. Thereafter, PBS solution of NOTA conjugated 4hD29 (around 30 µl, approximately 3 nmol) was added to the buffered ^68^Ga eluent.

The optimized labeling conditions included heating at 40°C for 10 minutes instead of being left at room temperature (Supplementary Figure 1C).

The crude product was purified using a NAP-5 column. The column was pre-equilibrated with 10 mL of PBS containing 10% ethanol, and then the reaction mixture was loaded onto the column. The NAP-5 column was eluted with fractions of 200 μl, 700 μl and 300 μl of PBS. The second fraction, containing the final product, was collected for purity control. The radiochemical purity (RCP) of the labeled nanobody was assessed using a high-performance liquid chromatography (HPLC, VWR Hitachi Chromaster 5110 pump) with UV- (Knauer 40D) and radiosignal (Bioscan B-FC-3300) detectors coupled in series. Analyte separation was accomplished utilizing a BIO Wide Pore C5 column sourced from Sigma Aldrich. The HPLC analysis followed this method: Milli-Q water with 0.1% trifluoroacetic acid (TFA) served as mobile phase A, while acetonitrile with 0.1% TFA served as mobile phase B. The elution gradient ranged from 20% to 80% B over 10 minutes at a flow rate of 1 ml/min, with UV detection at 280 nm. All chemicals were procured from Sigma Aldrich and used without prior purification.

*In vitro and in vivo stability of [^68^Ga]Ga-NOTA-4hD29*

The stability of the [^68^Ga]Ga-NOTA-4hD29 tracer was evaluated in formulation buffer (shelf life), *in vitro* in human plasma, and *in vivo* in rats. To assess stability in the formulation buffer, [^68^Ga]Ga-NOTA-4hD29 was incubated in PBS at room temperature for 30 or 60 minutes, and its radiochemical purity (RCP) was compared with that determined at the end of synthesis. Samples were analyzed by HPLC without prior processing.

For the assessment of tracer stability in plasma *in vitro*, samples were incubated in human plasma at a 4:1 plasma to tracer ratio at 37°C for 15 or 60 minutes. *In vivo* stability in rat plasma was investigated by blood sampling at 5, 30, or 60 minutes after intravenous administration of [^68^Ga]Ga-NOTA-4hD29.

Before radio-HPLC analysis, each plasma sample was treated with acetonitrile in a 1:1 ratio to precipitate proteins, followed by centrifugation at 16,000 g for 2 minutes using an Eppendorf 5415R centrifuge operating at 4°C. The resulting supernatant was filtered through a 0.2 μm nylon membrane (Acrodisc Syringe Filters, New York, United States) before being introduced into the HPLC system for detailed analysis, employing the previously mentioned HPLC methodology.

*Animal experiments*

Rats (n = 11, male, Sprague Dawley) were housed in pairs under standardized conditions, including a constant temperature of 25 °C, 50% relative humidity, and a 12-hour light/dark cycle. Food and water were provided ad libitum. Immunodeficient NMRI nu/nu mice (n=29; Taconic M&B, Ejby, Denmark) were housed in a pathogen-free facility and had free access to autoclaved water and pellet food.

In addition, one pig (n = 1; crossbred Yorkshire × Swedish Landrace × Hampshire; weight: 31.8 kg) was transported from a commercial farm to the imaging facility in accordance with local animal handling protocols.

*Access to human islets and generation of SC-islets for in vivo transplantation*

Human islets were obtained from the Nordic Network for Clinical Islet Transplantation (Rudbeck Laboratory, Uppsala University, Sweden) and cultured in CMRL1066 medium (Gibco, NY, USA) with the addition of 50 mmol/L L-glutamine (Sigma-Aldrich, MO, USA), 10% fetal calf serum and 5 U/mL Penicillin Streptomycin (Roche, Sigma-Aldrich).

H1 cells were cultured on recombinant human Laminin-521 (LN-521, BioLamina, Sundbyberg, Sweden) in mTeSR-Plus medium (STEMCELL Technologies, #100–0274/100–0275). Before induction, cells were seeded at a density of 200.000 per cm². Culture dishes were pre-coated with LN-521 and supplemented with 10 µM Y-27632 (ROCK inhibitor; STEMCELL Technologies, #72304) to enhance cell viability during plating. Differentiation was initiated 24 hours post-seeding.

Stages 1 to 4 (S1–S4) were conducted under adherent conditions. At substage 4:3, to ensure uniform aggregate formation, cells were enzymatically dissociated into a single-cell suspension and seeded to microwells (6-well AggreWell 400 plates, Stem Cell Technologies) at a density of 800–1,000 cells per microwell using the manufacturer’s recommended protocol. On the second day of Stage 5 (S5) culture, the SC-islets were transferred from the microwells to suspension culture on ultralow attachment plates (Corning, catalog no. CLS3471) placed on a rotator spinning at 95 r.p.m. Medium was changed daily throughout stages S1 to S5, and every 48 hours during stages S6 and S7.

*In vivo biodistribution in pig using PET*

*In vivo* biodistribution of [^68^Ga]Ga-NOTA-4hD29 was assessed in a healthy pig (n=1, Yorkshire x Swedish Landrace x Hampshire, weight 30 kg). The pig was anesthetized, ventilated, and monitored as described in detailed previously (2). placed in the PET/CT scanner lying on its back, ensuring that its abdomen was at the center of the Field of View (FOV). An attenuation CT scan was conducted using a 64-slice digital 4-ring system with a 198-mm axial FOV. The scan settings were as follows: 100 kV, 80–400 mA, noise index 10, rotation time of 0.5 seconds, full spiral acquisition, 3.75 mm slice thickness, pitch ratio of 0.98:1, and a reconstruction diameter of 50 mm.

Afterward, the pig received an intravenous injection of [^68^Ga]Ga-NOTA-4hD29 (0.8 MBq/kg), followed by a 90-minute dynamic PET scan over the pig’s abdomen. The scan protocol comprised 30 frames, including 12 frames of 10 seconds each, 6 frames of 30 seconds each, 5 frames of 2 minutes each, 5 frames of 5 minutes each, and 2 frames of 10 minutes each. Throughout the scan, arterial blood samples were collected at 5, 30, 60, and 90 minutes post-injection to assess the tracer's activity levels in both plasma and whole blood.

The PET images were reconstructed utilizing an iterative VPFX-S algorithm provided by GE Healthcare, employing 3 iterations, 16 subsets, a 3-mm postfilter, and a 256 × 256 matrix. After the PET scans, the pig was euthanized via intravenous administration of potassium chloride (KCl) while under deep anesthesia.

*Single cell transcription of DPP6 in pancreatic cells in a reference database*

The human tissue transcription of *DPP6*, encoding the DPP6 protein, was analysed from publicly available reference data *Tabula Sapiens*. The database contains single cell RNA sequencing (snRNAseq) of >1 million cells from most major organs from healthy human subjects (7-8). The single-cell transcription of *DPP6* was evaluated specifically in cells in the pancreas to investigate the specificity to the endocrine compartment.

*Immunohistochemistry of explanted islet xenografts*

The islet xenografts were fixed overnight in 4% PFA at 4˚C, followed by incubation in 15% sucrose in PBS for 2–3 h and then 30% sucrose in PBS overnight at 4˚C and frozen. Cryosections of SC-islets or grafts were prepared with a thickness of 10 μm. Cryosections were blocked with 3% donkey serum (Jackson Immunoresearch Laboratories, West Grove, PA, USA) in PBS at RT for 30 min followed by incubation overnight at 4°C with primary antibodies, guinea-pig anti-human insulin (dilatation 1:500; Fitzgerald, Acton, MA, USA), mouse anti-glucagon with Alexa Fluor 488-conjugate (dilution 1:500; Invitrogen Life Technologies) and Alexa-4hD29 (dilution 1:200). The following day, cryosections were washed and incubated for 1 hour at RT with the secondary antibody anti-guinea pig Alexa Fluor 647 (1:300; Jackson Immunoresearch Laboratories). Nuclei were counterstained with Hoechst (1:10,000; Thermo Fisher Scientific) and samples were mounted using Fluorescence Mounting Medium (Dako, North America Inc., Santa Clara, CA, USA). Imaging was acquired using a Zeiss LSM 780 confocal microscope (Carl Zeiss Microscopy GmbH).

**Supplementary Results**

*Transcription of DPP6 in pancreatic cells*

*DPP6* was almost exclusively present in endocrine cells in the pancreas (Supplementary Figure 18A-B). Among individual endocrine cell populations, histogram analysis demonstrated expression primarily in the alpha and beta-cells and minimal contribution from delta and PP cells (Supplementary Figure 18C).

**Declarations**

*Ethics approval and consent to participate*

All the performed procedures were in accordance with the ARRIVE guidelines for animal research. The animal experiments were ethically reviewed, authorized by the Animal Ethics Committee of the Swedish Animal Welfare Agency and carried out in accordance with the European Directive 2010/63/EEC and the institutional guidelines at Uppsala University (UFV 2007/724).

All human biological sample donors in Sweden provide informed consent that their donated tissues may be entered into a BioBank and used in medical research, pending review and approval by the Swedish Ethical Review Authority of individual studies. The use of human tissues and islets from Uppsala Biobank (registration #827) was approved by the Swedish Ethical Review Authority (2015/401, 2019/02790, 2024-00969) and were anonymized, collected, and treated according to local institutional and Swedish national rules and regulations. The use of human blood from the local biobank was approved by the Swedish Ethical Review Authority (2024-05050). The differentiation and use of iPSCs into islet-like cells was approved by the Ethics Committee of the Erasmus Hospital, Université Libre de Bruxelles, reference P2019/498.

*Consent for publication*

Not applicable.

*Availability of data and material*

The datasets used and/or analyzed during the current study are available from the corresponding authors on reasonable request.

*Competing interests*

OE is a part-time employee of Antaros Tracer AB. SJ and ACT are employees of InSphero AG, a company commercializing human islet microtissues and related services. B.Y. is a member of the management team of InSphero AG. DLE is a member of the Scientific Advisory Board of InSphero AG. The remaining authors declare that they have no competing interests related to the present study.

*Funding*

The study was funded by the Helmsley foundation (R-2207-05413) for DLE and OE, by the Walloon Region (Win4Excellence grant, GT4Health project, agreement number 2310145) to DLE, by the Barndiabetesfonden (JL and OE), Diabetesfonden, Science for Life Laboratory, the Swedish Research Council (2020-0231 and 2024-03659; OE, 2019-01415 and 2023-02221; OK), Swedish Cancer Society/ Cancerfonden) (24 3754 Pj; OE, 21 1519 Pj; OK, 24 3889 Pj; LRO), an EFSD/Lilly grant, StemTherapy (JL), ExoDiab and the Ernfors Family Fund (JL and OE).

*Authors' contributions*

SV, JL, LJIB, IV, LO, SJ, ACT, BY, OK and LRO performed experimental research, interpreted data and edited the manuscript. AK and AR-R conceptualised and designed different parts of the study, performed experimental research, interpreted data and wrote the manuscript. OE, JL and DLE conceptualized the study, interpreted the data and wrote the manuscript. OE is the guarantor of this work and, as such, had full access to all the data in the study and takes responsibility for the integrity of the data and the accuracy of the data analysis.

*Acknowledgements*

The corelab staff at Preclinical PET/MRI Platform (PPP) and Hedenstierna laboratory at Uppsala University are acknowledged for technical assistance. A. Bitzios at the Department of Medicinal Chemistry and Z. Li at the Department of Medical Cell Biology at Uppsala University are acknowledged for excellent technical assistance. We thank J. Capitaine, I. Millard, N. Belmahjoubi and N. Pachera (ULB Center for Diabetes Research, Université libre de Bruxelles, Brussels, Belgium) for their excellent technical support; and Dr Eugenia Martín-Vázquez and Dr Maria Lytrivi (ULB Center for Diabetes Research) for providing paraffin-embedded samples. Some of the data were presented as a poster abstract at the 84^th^ ADA annual meeting in 2024 and as an oral abstract at the 61^st^ EASD Annual Meeting in 2025.

**Supplementary References**

1.	Lytrivi M, Senée V, Salpea P, et al. DNAJC3 deficiency induces β-cell mitochondrial apoptosis and causes syndromic young-onset diabetes. Eur J Endocrinol. 2021;184:455-468.

2.	Cosentino C, Toivonen S, Villamil ED, et al. Pancreatic β-cell tRNA hypomethylation and fragmentation link TRMT10A deficiency with diabetes. Nucleic Acids Res. 2018;46:10302-10318.

3.	Fantuzzi F, Toivonen S, Schiavo AA, et al. In depth functional characterization of human induced pluripotent stem cell-derived beta cells in vitro and in vivo. Front Cell Dev Biol. 2022;10.

4.	Moore F, Colli ML, Cnop M, et al. PTPN2, a Candidate Gene for Type 1 Diabetes, Modulates Interferon-γ–Induced Pancreatic β-Cell Apoptosis. Diabetes. 2009;58:1283.

5.	Schneider CA, Rasband WS, Eliceiri KW. NIH Image to ImageJ: 25 years of image analysis. Nature Methods 2012 9:7. 2012;9:671-675.

6.	Demine S, Garcia Ribeiro R, Thevenet J, et al. A nanobody-based nuclear imaging tracer targeting dipeptidyl peptidase 6 to determine the mass of human beta cell grafts in mice. Diabetologia. 2020;63:825-836.

7.	Tabula Sapiens Consortium*; Jones RC, Karkanias J, Krasnow MA et Al. The Tabula Sapiens: A multiple-organ, single-cell transcriptomic atlas of humans. Science. 2022 May 13;376(6594):eabl4896.

8.	https://tabula-sapiens.sf.czbiohub.org, accessed 07-JUN-2025

**Supplementary Figures**

**Supplementary Figure 1.** Schematic illustration of the original DPP6 targeting sdAb (A), chelator conjugation to generate the precursor peptide (B) as well as Gallium-68 radiolabeling conditions to form the PET tracer [^68^Ga]Ga-NOTA-4hD29 (C).

**Supplementary Figure 2.** Representative dithizone staining microscopy images for the islet preparations with 24%, 43%, or 90% of beta cell purity, with islets (zinc-containing cells) appearing red and exocrine cells in yellow.

**Supplementary Figure 3. Characterization of Alexa-4hD29 binding to human beta cells.** (**A**) HeLa cells were incubated with different concentrations of Alexa-4hD29 (100 or 6.25 μg/ml). Images were obtained by fluorescence microscopy. Images are representative of three independent experiments. (**B**) EndoC-βH1 cells were incubated with different concentrations of Alexa-4hD29 (100, 50, 25, or 6.25 μg/ml). Images were obtained by fluorescence microscopy. Images are representative of three independent experiments. (**C**) EndoC-βH1 cells were silenced for DPP6 by using different concentrations of DPP6-specific siRNA (7.5, 15, or 30 nM). After 48 h of recovery the cells were fixed and incubated with Alexa-4hD29. Images were obtained by fluorescence microscopy. Images are representative of three independent experiments. (**D**) DPP6 and GAPDH protein levels were analysed by immunoblot. Results are means ± SEM of three independent experiments. Bands were quantified by densitometry and corrected by GAPDH. Results are presented as fold-variation compared to untreated cells, considered as 1. *p < 0.05, **p < 0.01, and ***p < 0.001 vs. untreated cells. One-way Anova. **E**) DPP6 vs DAPI relative intensity comparison. Results are presented as fold-variation compared to untreated cells, considered as 1. **p < 0.01, and ***p < 0.001 vs. untreated cells. One-way Anova. (**F**) Dispersed iPSC-derived islet-like cells were incubated with Alexa-4hD29 plus insulin and glucagon. Images were obtained by fluorescence microscopy. Images are representative of three independent experiments. Scale is 200 μm.

**Supplementary Figure 4. Autofluorescence of Human islet microtissues (hIsMTs).**

HIsMTs were incubated at 37ºC in the absence of Alexa-4hD29. Cells were then washed three times with PBS and kept in culture at 37ºC at different time-points (0, 24h, 48h, 72h, or 96h). Images are representative of a single islet organoid. Scale is 50 μm.

**Supplementary Figure 5.** **Alexa-4hD29 enters the beta cell lysosomal pathway.** Living EndoC-βH1 (**A**) or iPSC-derived islet-like cells (**B**) were incubated at 37ºC with 50 μg/ml Alexa-4hD29 for 1h. Cells were the washed three times with PBS and kept in culture 1h or 4h at 37ºC. After this, cells were fixed, and preparations were incubated with antibodies recognizing LAMP1, RAB7, or RAB11. Preparations were then mounted with DAPI for widefield fluorescence microscopy. Scale is 200 μm. Results are means ± SEM of three independent experiments.

**Supplementary Figure 6.** **Red blood cells´ autofluorescence.** Human Multi-tissue Tissue MicroArrays corresponding to 59 human tissues from different locations obtained from different donors were incubated or not at 4ºC overnight with Alexa-4hD29 and insulin antibody, or with PBS. After incubation with the corresponding secondary antibody, slides were mounted with DAPI and representative images were analysed by fluorescence microscopy. Images on the left correspond to consecutive slide cuts incubated with PBS; on the right, consecutive slide cuts incubated with antibodies. The experiment was performed twice and the selected tissues are shown. Scale is 200 μm.

**Supplementary Figure 7. DPP6 signal is specific to human islets. (a) Human** Multi-tissue Tissue MicroArrays corresponding to 59 human tissues from different locations obtained from different donors were incubated at 4ºC overnight with DPP6 and insulin antibodies. After incubation with the corresponding secondary antibodies, slides were mounted with DAPI and representative images were analysed by fluorescence microscopy. The experiment was performed twice, and selected tissues are shown. Scale is 200 μm.

**Supplementary Figure 8. DPP6 signal is specific to primate beta cells.** (**A**) Mouse pancreas, (**B**) human iPSC-derived islet-like cells, (**C**) Cynomolgus macaque pancreas, or (**D**) pig pancreas were incubated at 4ºC overnight with DPP6 and insulin antibodies. After incubation with the corresponding secondary antibodies, slides were mounted with DAPI and representative images were analysed by fluorescence microscopy. The experiment was performed twice. Scale is 200 μm.

**Supplementary Figure 9.** Representative LC-MS trace of the coupling reaction between the 4hD29 nanobody and *p*-SCN-Bn-NOTA (ratio of 1:12) after 22 hours of incubation at room temperature.

**Supplementary Figure 10.** Representative LC-MS trace for the collected fraction 4 showing the successful purification of the NOTA-conjugated 4hD29 using a PD-10 desalting column.

**Supplementary Figure 11.** Representative LC-MS trace for the collected fraction 6 showing the successful purification of the NOTA-conjugated 4hD29 using a PD-10 desalting column.

**Supplementary Figure 12.** The stability of [^68^Ga]Ga-NOTA-4hD29 in formulation buffer, *in vitro* in human plasma, or *in vivo* in rat plasma, expressed as the percentage of intact tracer over time.

**Supplementary Figure 13.** HPLC radio-chromatograms showing the stability of [^68^Ga]Ga-NOTA-4hD29 *in vitro* in human plasma after 15 minutes (A) or 60 minutes (B) incubation.

**Supplementary Figure 14.** Representative HPLC radiochromatograms showing the stability of [^68^Ga]Ga-NOTA-4hD29 *in vivo* in rat plasma after 5 minutes (A), 30 minutes (B), or 60 minutes (C) after tracer injection.

**Supplementary Figure 15.** Representative autoradiograms demonstrating low or negligible binding of [^68^Ga]Ga-NOTA-4hD29 to spleen, heart (myocardium), liver and lung.

**Supplementary Figure 16.** *Ex vivo* biodistribution in rat demonstrating the retention of [^68^Ga]Ga-NOTA-4hD29, expressed as SUV (A). Representative PET/MRI images illustrating the biodistribution of [^68^Ga]Ga-NOTA-4hD29 *in vivo* in rat at 2, 60, or 120 minutes post- injection (B). Quantification of the *in vivo* uptake in rats as examined by PET imaging for up to 120 minutes (C). Br: brain, Li: liver, Lu: lungs, Ki: kidney.

**Supplementary Figure 17.** Biodistribution of [^68^Ga]Ga-NOTA-4hD29 in pig visualized through representative dynamic PET/CT scanning at 15, 30, and 60 minutes post-tracer injection. (A). Quantification of PET uptake data as standardized uptake values (SUV) (B) Li: liver, Lu: lungs, Ki: kidney.

**Supplementary Figure 18.** Single-cell RNA sequencing data from human pancreas, showing annotated clusters of distinct pancreatic and immune cell types (A). UMAP visualization of *DPP6* expression, with a gradient from low (green) to high (purple) transcription (B). Histogram showing *DPP6* expression levels in individual endocrine subtypes (alpha, beta, delta, PP cells) (C).

**Supplementary Tables**

**Supplementary Table 1.** List of primary and secondary antibodies used.

| Immunoblot |  |  |  |  |
| --- | --- | --- | --- | --- |
| Antibody | Supplier | Reference | Dilution | RRID |
| Peroxidase- conjugated donkey anti-rabbit IgG | Jackson ImmunoResearch, Ely, UK | 715-036-152 | 1:5000 | AB_2340590 |
| Peroxidase- conjugated donkey anti-mouse IgG | Jackson ImmunoResearch | 711-036-150 | 1:5000 | AB_2340773 |
| Human DPP6 Antibody | R&D System, Abingdon, UK | MAB2360 | 1:1000 | AB_2093854 |
| Anti-GAPDH | Treveigen, Gaithersburg, MD, USA | 2275-PC-020 | 1:5000 | N/A |
|  |  |  |  |  |
|  |  |  |  |  |
| Immunocytochemistry/Immunohistochemistry |  |  |  |  |
| Antibody | Supplier | Reference | Dilution/concentration | RRID |
| Alexa Fluor® 488 AffiniPure Donkey Anti-Rabbit IgG (H+L) | Jackson ImmunoResearch | 711-545-152 | 1:500 | AB_2313584 |
| Alexa Fluor® 647 AffiniPure Donkey Anti-Rabbit IgG (H+L) | Jackson ImmunoResearch | 711-605-152 | 1:500 | AB_2492288 |
| Alexa Fluor® 488 AffiniPure Donkey Anti-Mouse IgG (H+L) | Jackson ImmunoResearch | 715-545-151 | 1:500 | AB_2341099 |
| Rhodamine Red™-X (RRX) AffiniPure Donkey Anti-Mouse IgG (H+L) | Jackson ImmunoResearch | 715-295-151 | 1:500 | AB_2340832 |
| Rhodamine Red™-X (RRX) AffiniPure Donkey Anti-Goat IgG (H+L) | Jackson ImmunoResearch | 705-295-147 | 1:500 | AB_2340423 |
| Alexa Fluor® 488 AffiniPure Donkey Anti-Guinea Pig IgG (H+L) | Jackson ImmunoResearch | 706-545-148 | 1:500 | AB_2340472 |
| Glucagon Mouse | Sigma, Saint-Louis, MO, USA | G2654 | 1:200 | AB_259852 |
| Guinea pig anti-human insulin | Agilent, Santa Clara, CA, USA | A056401-2 | RU | AB_2617169 |
| LAMP1 (D2D11) XP | Cell Signaling, Leiden, The Netherlands | 9091 | 1:100 | AB_2687579 |
| Rab7 (D95F2) XP | Cell Signaling | 9367 | 1:100 | AB_1904103 |
| Rab11 (D4F5) XP | Cell Signaling | 5589 | 1:100 | AB_10693925 |
| Human DPP6 Antibody | R&D System, Abingdon, UK | MAB2360 | 1:100 | AB_2093854 |
| Alexa-4hD29 | sdAb and conjugation generated in-house | N/A | - 50 μg/ml for 1-2h incubation in living cells (37ºC)  - 100-200 μg/ml for overnight incubation (4ºC) | N/A |
|  |  |  |  |  |
| N/A = Not available  RU = Ready to use |  |  |  |  |

**Supplementary Table 2.** Organ and whole-body human predicted dosimetry of [^68^Ga]Ga-NOTA-4hD29, extrapolated from rat *in vivo* biodistribution data. Text in red indicates limiting annual doses and potential dosing limits based on national Swedish regulations.

| **Tissue** | **Absorbed dose (mGy/MBq)** | **Annual regulatory limit (mGy)** | **Annual dosing limit (MBq)** |
| --- | --- | --- | --- |
| **Adrenals** | 0.00228 | 150 | 65789 |
| **Brain** | 0.0011 | 150 | 136364 |
| **Breasts** | 0.000341 | 150 | 439883 |
| **Gallbladder wall** | 0.00494 | 150 | 30364 |
| **LLI wall** | 0.00633 | 150 | 23697 |
| **Small intestine** | 0.00302 | 150 | 49669 |
| **Stomach** | 0.00144 | 150 | 104167 |
| **ULI wall** | 0.00291 | 150 | 51546 |
| **Heart** | 0.0113 | 150 | 13274 |
| **Kidneys** | 0.133 | 150 | 1128 |
| **Liver** | 0.00429 | 150 | 34965 |
| **Lungs** | 0.00334 | 150 | 44910 |
| **Muscle** | 0.00258 | 150 | 58140 |
| **Ovaries** | 0.0048 | 50 | 10417 |
| **Pancreas** | 0.0117 | 150 | 12821 |
| **Red Marrow** | 0.00397 | 50 | 12594 |
| **Osteogenic cells** | 0.00455 | 50 | 10989 |
| **Skin** | 0.000714 | 150 | 210084 |
| **Spleen** | 0.0042 | 150 | 35714 |
| **Testes** | 0.00335 | 50 | 14925 |
| **Thymus** | 0.000565 | 150 | 265487 |
| **Thyroid** | 0.000305 | 150 | 491803 |
| **Urinary Bladder** | 0.411 | 150 | 365 |
| **Uterus** | 0.0106 | 150 | 14151 |
| **Total Body** | 0.00304 | 150 | 49342 |
|  | **mSv/MBq** | **mSv limit** | **MBq limit** |
| **Effective Dose** | 0.0245 | 10 | 408 |
